# Inflammatory Links Between High Fat Diets and Diseases

**DOI:** 10.3389/fimmu.2018.02649

**Published:** 2018-11-13

**Authors:** Yehui Duan, Liming Zeng, Changbing Zheng, Bo Song, Fengna Li, Xiangfeng Kong, Kang Xu

**Affiliations:** ^1^Hunan Provincial Key Laboratory of Animal Nutritional Physiology and Metabolic Process, Key Laboratory of Agro-ecological Processes in Subtropical Region, Institute of Subtropical Agriculture, Chinese Academy of Sciences, Hunan Provincial Engineering Research Center for Healthy Livestock and Poultry Production, Scientific Observing and Experimental Station of Animal Nutrition and Feed Science in South-Central, Ministry of Agriculture, National Engineering Laboratory for Pollution Control and Waste Utilization in Livestock and Poultry Production, Changsha, China; ^2^Science College of Jiangxi Agricultural University, Nanchang, China; ^3^Guangdong Provincial Key Laboratory of Animal Nutrition Regulation, South China Agricultural University, Guangzhou, China

**Keywords:** high-fat diets, inflammation, disease, mechanisms, drug therapy

## Abstract

In recent years, chronic overnutrition, such as consumption of a high-fat diet (HFD), has been increasingly viewed as a significant modifiable risk factor for diseases such as diabetes and certain types of cancer. However, the mechanisms by which HFDs exert adverse effects on human health remains poorly understood. Here, this paper will review the recent scientific literature about HFD-induced inflammation and subsequent development of diseases and cancer, with an emphasis on mechanisms involved. Given the expanding global epidemic of excessive HFD intake, understanding the impacts of a HFD on these medical conditions, gaining great insights into possible underlying mechanisms, and developing effective therapeutic strategies are of great importance.

## Introduction

At present, obesity has reached epidemic proportions. It develops from an imbalance of energy homeostasis and contributes dramatically to the global disease burden, predisposing individuals to chronic diseases such as type 2 diabetes mellitus (T2DM), cardiovascular disease (CVD), and certain types of cancer (1, 2). Excessive consumption of high fat diets (HFDs) has undoubtedly exacerbated the obesity epidemic and the development of obesity-related metabolic disorders (3, 4). Despite substantial work demonstrating that obesity develops from an imbalance between energy intake and energy expenditure, the underlying mechanisms for the detrimental effects of HFDs seem to be more complicated than the simple concept of energy imbalance (5). In this context, a better understanding of the pathogenesis of HFD-driven metabolic disorders may help to reduce the disease burden worldwide. This review will elaborate on the precise pathology and etiology of diseases induced by a HFD, and will evaluate the possibility of therapeutic targeting of free fatty acids (FFAs) and low-grade systemic inflammation to reduce HFDs-stimulated diseases.

## HFDs induce a systemic chronic low-grade inflammation

Following the ingestion of HFDs, inflammation develops in the central nervous system (CNS) including the hypothalamus and in the peripheral tissues including the liver, adipose tissue, skeletal muscle, and intestine (6). Concerning the development of chronic systemic inflammation, alterations in the gut microbiota triggered by HFDs and direct effects of FFAs on intestinal cells may be the first step (7). Evidence to support this hypothesis is that germ-free mice exhibited neither obesity nor upregulation of intestinal TNF-α level compared to conventionalized mice when fed a HFD, whereas reconstitution of the gut microbiota from obese mice in the germ-free mice produced an increase in body fat (8). Furthermore, after comparing microbiota of obese vs. lean subjects, a recent study suggested that individuals with high bacterial richness were associated with less significant adiposity and inflammation than low bacterial richness subjects (9), which is line with HFD-induced inflammation and decrease of bacterial diversity. In particular, increased levels of *Firmicutes* and a reduced relative abundance of *Bacteroidetes* were observed in both humans and animals following HFD intake. Shifts in gut microbiota populations activate Toll-like receptor (TLR) signaling pathway, leading to increased intestinal permeability to endotoxins [such as lipopolysaccharides (LPS)] and thus promoting the translocation of LPS to the circulation (10–12). In addition, increased amounts of FFAs present in HFDs may directly act on intestinal cells. Therefore, elevated release of LPS and/or increased FFAs levels led to elevated production of pro-inflammatory cytokines [i.e., interleukin (IL)-1β, IL-6, and tumor necrosis factor (TNF)-α] in the gut (13–17). The second step may consist in increased delivery of intestinal LPS, pro-inflammatory cytokines, and FFAs into the systemic circulation and portal circulation, thus leading to a systemic low-grade inflammation (15, 18). Elevated plasma FFAs and LPS can upregulate the expression of TLRs in circulating macrophages, enabling macrophages to be activated (M1 phenotype), which in turn produce proinflammmatory cytokines (11, 12).

Before the onset of obesity, these factors in the circulating system triggers inflammatory pathways in the brain. More especially, elevated FFAs and cytokines first activate hypothalamic IκB kinase β (IKKβ)/nuclear factor of kappa B (NF-κB) signaling directly or indirectly through the following two ways: (i) through activating TLR located at cellular surface; (ii) through inducing various cellular stresses including oxidative stress and endoplasmic reticulum stress (ERS) in the hypothalamus (19–21). Hence, activated IKKβ/NF-κB signaling blunts central leptin and insulin sensitivity and initiates gene expression of inflammatory response molecules in the hypothalamus (19, 22, 23). Meanwhile, activated inflammatory macrophages (M1) in plasma can reach the adipose and muscular tissues, pancreatic islets, and blood vessels, leading to peripheral inflammation (12, 24). Notably, accumulation of CD8^+^ T-cells in the adipose tissue contributes to M1 macrophage recruitment in the adipose tissue (25). In addition, under HFD feeding stress the adipose tissue fails to store the excess lipids, which are thereafter deposited into other tissues including the liver, pancreas, skeletal muscle, and blood vessels (24). Ectopic lipid accumulation contributes additionally to the expression of proinflammatory mediators and the recruitment of M1 macrophages, thus aggravating systemic inflammation (26, 27). Moreover, the liver is also exposed to relatively high concentrations of different mediators (i.e., LPS, proinflammatory cytokines, and FFAs) released by the gastrointestinal tract (15). These mediators lead to accumulation of Natural killer T (NKT) cells and activation of Kupffer cells in the liver, thereby contributing to hepatic and systemic inflammation (28).

**Figure 1:**
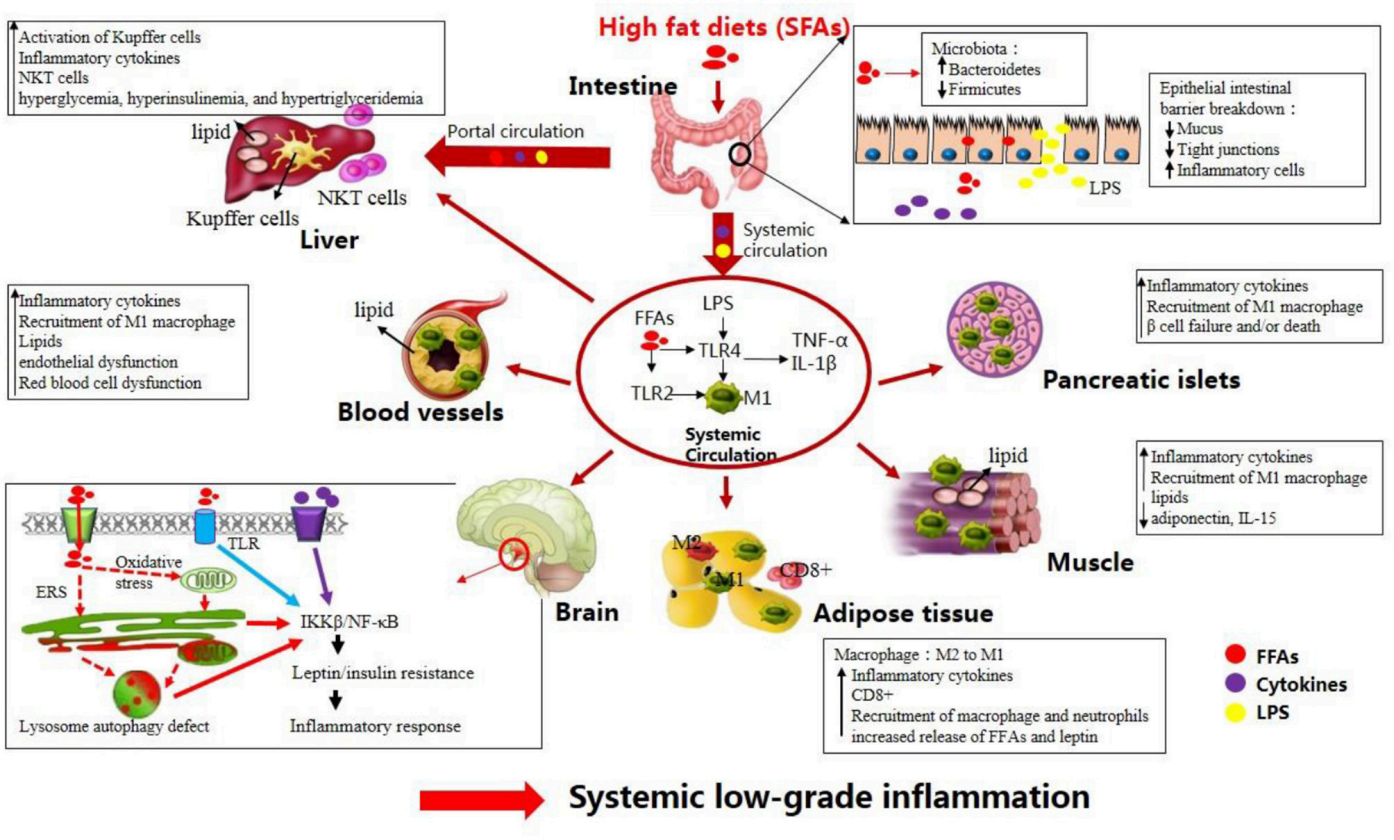
High-fat diets (HFDs) induce metabolic inflammation throughout the organism. The levels of endotoxins (e.g., LPS), circulating free fatty acids, and inflammatory mediators are increased in response to HFDs, resulting in low-grade systemic inflammation and altered homeostasis in many organs (see text).

**Figure 2:**
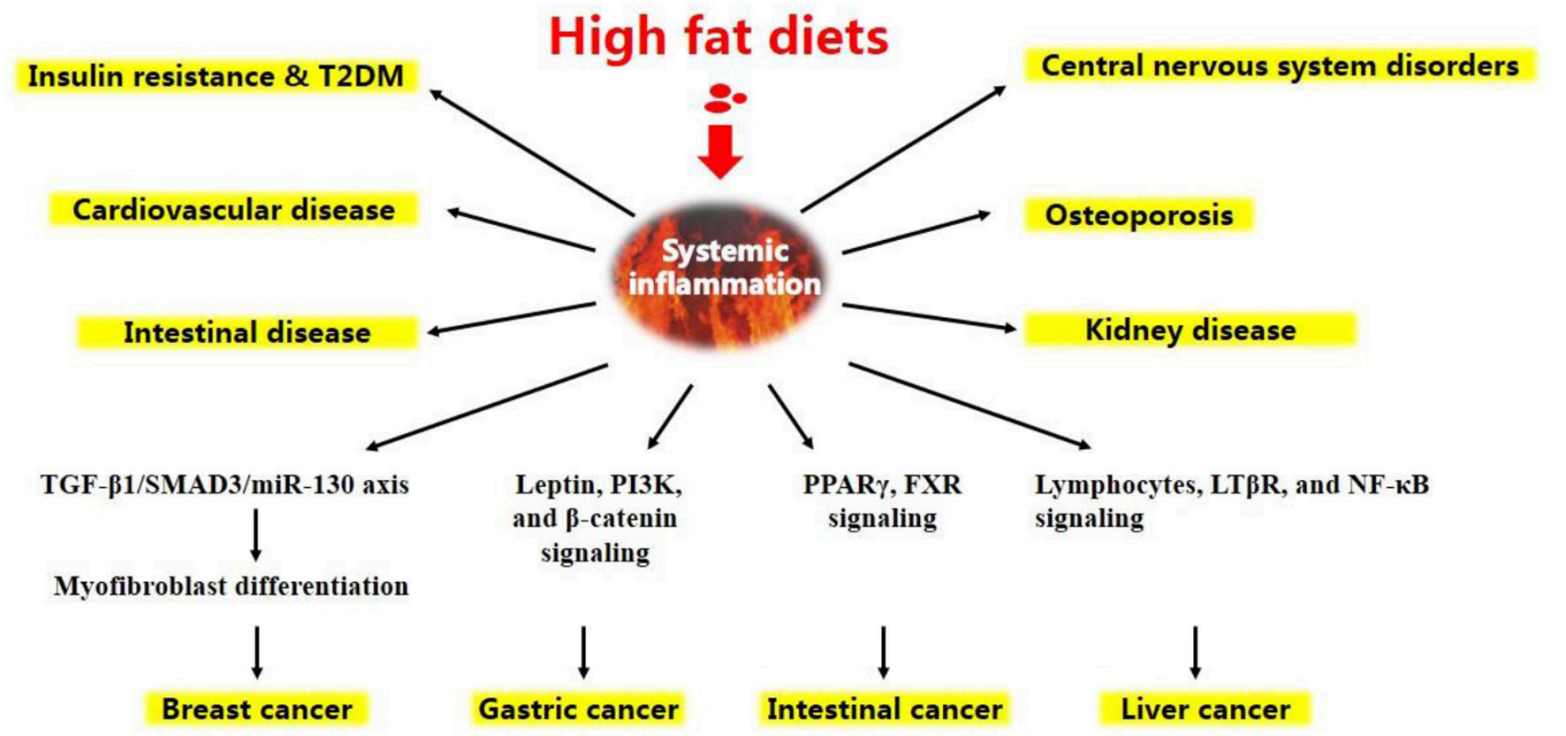
Chronic diseases and certain types of cancer are induced by high-fat diets.

Altogether, under HFD conditions, a complicated network of signals interconnecting several organs acts in synergy to elicit a low-grade systemic inflammation (Figure 1). HFD-related inflammation leads to the failure of adipocytes to effectively remove circulating FFAs, and is pivotal to disease progression and the development of complications, such as T2DM, CVD, liver disease, atherosclerosis, and certain types of cancer (Figure 2, discussed in detail below).

## HFD-induced diseases

### Insulin resistance and T2DM

Insulin resistance is the condition where the body fails to respond appropriately to circulating insulin, leading to the impaired systemic glucose clearance and uptake in several tissues including the adipose tissue, liver, and muscle (29). Exposure to a HFD often results in insulin resistance and pancreatic β cell dysfunction. For example, after only 3 days of HFD feeding, systemic insulin resistance, and glucose intolerance developed in mice (30). Other studies have also shown that mice fed a HFD for several weeks exhibited increased glucose intolerant and impaired insulin sensitive, reflecting insulin resistance and initiating β cell compensation (31, 32).

Although not fully explored, current evidence has established causative and correlative links between HFD-induced inflammation and the pathogenesis of insulin resistance (27, 33). Under HFD conditions, insulin secretion from β cells and peripheral insulin action can be impaired by hypothalamic inflammation (34). Proinflammatory cytokines produced by β cells themselves due to macrophages accumulation in islets may further block β cell function (35). In addition, HFDs can activate 12- lipoxygenase activity in β cells through cytokines, FFAs, and increased blood glucose, thus increasing the production of 12-hydroxyeicosatetraenoic acid (12-HETE). 12-HETE promotes oxidative stress and blunts Nrf2 function, eventually leading to β cell apoptosis and glucose intolerance (36). Therefore, HFDs lead to an increase in insulin release from β cells and thus hyperinsulinemia. In adipose tissue, hyperinsulinemia combined with an inflammatory response promotes lipolysis, thus enabling FFAs and glycerol to flow to the liver and to promote gluconeogenesis. At the same time, elevated insulin concentrations increases muscle glycolysis and lactate production, which is released into the circulation and can be used as a substrate for gluconeogenesis in the liver (37). Subsequently, increased gluconeogenesis enhances glucose production in the liver and thereby leads to systemic insulin resistance (38). As a consequence, T2DM ensues due to systemic insulin resistance and relatively diminished insulin secretion from pancreatic β cells (29). In addition, recent studies indicated that the A2b adenosine receptor (A2bAR), an established mediator of inflammation, control pancreatic dysfunction in HFD-induced obesity (39). In particular, macrophage A2bAR has been recently identified as an important mediator of HFD-induced hallmarks of T2DM (40, 41). Therefore, regulation of macrophage A2bAR, either by genetic manipulations of monocytes or pharmacologically, may be a promising therapeutic approach. Taken together, chronically overconsumption of HFDs is mechanistically associated with the development of insulin resistance and T2DM.

### Cardiovascular disease

Although some controversy still exists, an increasing number of studies have highlighted the crucial effects of HFDs on the development of CVD (42, 43). For instance, administration of HFDs (20 g of fat/100 g of diet) to adult male rats for 30 days led to simultaneous increase in nitric oxide (NO) production and oxidative stress, causing the augmentation of infarct size after myocardial infarction (43). Low-grade systemic inflammation related to a HFD resulted in cardiovascular complications partially through infiltration of adipose and vascular tissues by effector T cells (44). In support of this notion, a cohort of 1,172 subjects was selected and stratified in three groups (i.e., lean, overweight, and obese). Results showed that the blood of overweight and patients with obesity had elevated numbers of CXCR3^+^ effector memory T cells (a pro-inflammatory effector memory-like phenotype) compared to lean subjects. Similar phenotype was also found in mice. Upon further investigation, the same group found that this differentiation of CXCR3^+^ effector memory T cells was partially mediated by the PI3K p110δ-Akt signaling pathway (44). Apart from infiltration of adipose and vascular tissues by effector T cells, the vascular endothelial dysfunction may also exert a role in the onset and propagation of CVD induced by a HFD (45, 46). Evidence for this hypothesis is provided by observations that mice fed with a HFD rich in lard exhibited an inhibition of the L-arginine-NO pathway in red blood cells (RBCs) of mice (47). RBCs-produced NO can contribute to the intravascular NO pool and suppression of platelet aggregation, thus regulating vascular homeostasis (48). Hence, decreasing NO bioavailability contributes to CVD via impairing vascular endothelial function. The adverse effects of HFD on endothelial function may be mediated independently by hyperglycemia, insulin resistance, and FFAs consequent to a HFD (49). Analogous to endothelial dysfunction, RBCs dysfunction takes place early during HFD-induced obesity and may contribute to the development of CVD (50). More especially, mice fed a 60% HFD for 12 weeks displayed a marked increase in the level of chemokines, which were bound to RBC via Duffy antigen receptor for chemokines. Moreover, RBCs from HFD-fed mice showed elevated cholesterol content and membrane phosphatidylserine externalization, accompanied by increased phagocytosis of RBCs by macrophages *in vitro* and by the spleen *in vivo*, thus contributing to atherosclerosis (50). Altogether, a HFD may promote the development of CVD via infiltration of adipose and vascular tissues by effector T cells, endothelial dysfunction and RBCs dysfunction.

### Intestinal diseases

A HFD-induced intestinal inflammation contributes to not only a systemic low-grade inflammation but localized tissue dysfunction (51, 52). For example, HFD feeding can exacerbate chemically-induced dextran sodium sulfate colitis by increasing pro-inflammatory cytokines (53–55) and can increase mucosal tissue damage in mouse models of ileitis (Mdr1a^−/−^ mice) (56, 57) and spontaneous colitis (Muc2^−/−^, TNF^Δ*ARE*^ mice) (58). Consistently, human studies have involved HFD consumption with an increased risk of functional bowel symptoms (59), such as inflammatory bowel diseases (IBD) (60). The increased incidence of IBD such as Crohn's disease (CD) in developed countries parallels escalating consumption of fat (60, 61). A HFD promotes the development of CD partially via promoting gut inflammation and microbiome perturbations. Previous studies have shown that a HFD increased intestinal permeability, promoted intestinal mucosa dysbiosis, and altered microbiota composition with a profile characterized by the expansion of pro-inflammatory *Firmicutes* and *Proteobacteria* and the reduction of protective bacteria such as *Bacteroidetes*. These effects triggered by a HFD were accompanied by downregulated expression of PPARγ and GPR43. Thus, the activation of PPARγ signaling and GPR43 receptor pathway could be viewed as an effective strategy to treat CD patients (62, 63). Moreover, after intestinal inflammation, intestinal morphofunctional rearrangement of the enteric nervous system occurs in the murine model of HFD-induced obesity, contributing to the pathophysiology of digestive motor abnormalities (64, 65). Recent studies further report that excitatory tachykiniergic pathways, which can be regulated by A2bAR, are involved in the pathogenesis of colonic dysmotility induced by intestinal macrophages in HFD-induced obesity (16, 17).

### Osteoporosis

Osteoblasts and adipocytes are derived from the same mesenchymal stem cell (MSC) source (66), leading to interactions between osteoclastogenesis and adipogenesis that can be affected by dietary fat intake (67). Epidemiological evidence has suggested that consuming HFDs promotes obesity, which is closely associated with lower bone mineral density (BMD) and a higher risk of osteoporotic fractures (68–70). Similar observations have also been observed in animal studies. Mice given HFDs (21.1% fat) for 13 weeks had lower bone mass, which was associated with oxidative stress (71). Likewise, HFDs (45% energy as fat) for 14 weeks led to decreased trabecular bone volume and trabecular number in the proximal tibia (72). These results suggest that HFDs affect adversely bone quantity. In contrast, other human and animal studies have shown that HFDs induced higher BMD because of elevated mechanical loading (73, 74). Recently, it has been reported that short-term consumption (8 weeks) of HFDs increased bone mass via elevated mechanical loading; however, long-term consumption (16 and 24 weeks) of HFDs exerted negative effects on bone mass (75). Therefore, it raises the possibility that HFDs initially augment bone deposition because of elevated mechanical demand but eventually impairs bone formation and turnover due to metabolic dysregulation (76, 77).

Mice received HFDs (60 kcal% fat) for 6 weeks before challenge with B16F10 melanoma cells exhibited elevated bone marrow (BM) adipocytes compared with mice received with normal diets. The increased presence of adipocytes largely changes the hematopoietic stem cell niche in the BM microenvironment and sets up a niche for tumor cells, increasing their proliferation by IL-6 and Janus Kinase 2 (JAK2). Moreover, cancer cells interact with fat in the BM facilitates the accumulation of osteoclasts in the BM by expressing osteopontin. Therefore, the IL-6-JAK2-osteopontin axis is a critical signaling pathway for building the metabolic tumor niche in the context of a HFD-induced obesity (78). In addition, HFD can also regulate bone deposition via the hormone leptin secreted by HFD-induced adiposity (79). Taken together, HFDs regulate bone metabolism and lead to osteoporosis partially through proinflammatory cytokines (IL-6) and adipokines (leptin).

### Chronic kidney diseases

A strong association between intake of SFAs, hyperalbuminuria and kidney dysfunction was observed in a cross-sectional study including more than 19,000 adults > 45 years of age (80). Increasing studies using rodent models have address potential mechanisms for the development of kidney disease driven by HFDs. Rats fed HFDs (35% kcal from fat) for 10 weeks developed proteinuria, accompanied by increased inflammatory markers (TNF-α and NF-κB) and oxidative stress markers (NADPH oxidase) in the renal cortex (81). Another study using HFD-fed Sprague-Dawley rats (58% fat-derived calories, 6 weeks) has demonstrated similar molecular and metabolic effects (82). The effect of hyperlipidemia-driven inflammation on the development of kidney dysfunction was further demonstrated in Apo E^−/−^ mice fed HFDs for 4 weeks (83). These mice displayed greatly increased levels of TNF-α and IL-6, inducing renal inflammation, mesangial cell proliferation, and the progression of proteinuria. However, these effects were strongly attenuated in mice after treatment with anti-IL-6 receptor antibody. Upon further investigation, studies found that the activity of proinflammatory cytokines and profibrotic growth factors may be enhanced by renal sterol-regulatory element binding protein (SREBP1). Mice fed HFDs (60% fat) became obese, hyperinsulinemic, and hyperglycemic, associated with increased levels of SREBP1/2 (84). More importantly, deletion of SREBP1 in the mice blocked renal inflammation and proteinuria (85). These data suggest that a SREBP1-enhanced renal inflammation may represent a mechanism for the development of HFD-induced chronic kidney diseases.

### Central nervous system disorders

The neuroinflammation has been observed in brain structures such as the hypothalamus, hippocampus, amygdala, cortex, and brainstem, increasing the incidence of CNS pathologies such as cognitive impairments (86), behavioral abnormalities (such as depressive- and anxiety-like behaviors) (87), Alzheimer's disease (AD) (88). For instance, after 14 weeks feeding of HFDs (40% energy from fat), mice exhibited altered brain insulin signaling and cognitive functions (89). Without induction of obesity, HFDs (60% fat calories, 10 weeks) via alterations in gut microbiota can decrease memory and augment anxiety and stereotypical behaviors in mice (90). Long-term (16 months) exposition to HFDs has been shown to favor the deposition of amyloid beta (Aβ) peptide in the brain of mice, which is associated with the incidence of AD (91). These data underscore the strong effects of a HFD-induced changes on brain damage.

HFD-induced cognitive deficits may be associated with oxidative stress in the brain. Total levels of reactive oxygen species, superoxide, and peroxynitrite were significantly increased in mice fed HFDs (45% kcals from fat) compared to mice fed a control diet (10% kcals from fat) (92). Other studies also found elevated oxidative stress in HFD-fed mice which show clear cognitive impairment (86, 93). These studies highlight a role for oxidative stress in HFD-induced cognitive impairment. Unlike the underlying mechanisms for the induction of cognitive impairment, HFD promotes the development of anxiety-like behavior probably via upregulated expression of IL-1β in the amygdala (86) and changes in the GABAergic neurotransmission within the dorsomedial hypothalamus (94). In addition, HFD dramatically promotes the progression of AD-like pathology via elevation of cerebral amyloid deposition and oxidative stress, as demonstrated by previous studies (95). In particular, intake of HFDs results in peripheral insulin resistance and obesity, resulting in the disruption of the blood-brain barrier. Therefore, the transport of glucose and insulin to the CNS is limited, thus decreasing insulin signaling in the CNS. Hence, HFD-triggered peripheral hyperinsulinemia to some extent can initially provokes insulin deficiency in the brain, leading to lower expression of insulin degrading enzyme and less degradation (accumulation) of Aβ (96). At the same time, HFD-induced oxidative stress further contributes to impaired insulin signaling, thus increasing GSK-3β levels and Tau hyperphosphorylation. Increased Aβ levels and Tau hyperphosphorylation give rise to neurodegeneration and diminished synaptic plasticity, respectively, thus leading to the incidence of AD (89). Taken together, a HFD-induced damage to the brain is triggered by a number of mediators including inflammation, changes to integrity of blood-brain barrier, insulin resistance, and oxidative stress. These findings have important implications for better understanding how HFD may potentially induce brain dysfunction and the development of neurodegenerative disorders such as AD.

### Liver cancer

The current Western lifestyle will increase the risk of fatty liver disease. Non-alcoholic steatohepatitis (NASH) can be developed from fatty liver disease (steatosis) and can proceed to fibrosis, cirrhosis, and even hepatocellular carcinoma (HCC). Therefore, to better understand how dietary-induced metabolic changes contribute to the development of hepatic inflammation and NASH, a recent study used a HFD-induced obese murine model of NASH progression over 16 weeks (28). After 16 weeks, HFDs induced an obese and inflammatory phenotype in mice. More importantly, mice fed HFDs exhibited increased infiltration of NKT-cells and clusters of differentiation CD8^+^ T-cells in the liver. To further confirm a role for NKT cells and CD8^+^ T-cells, the same group used CD1d knockout (KO) and mAb mice experiments, respectively. Notably, CD1dKO mice have no functional NKT cells. After 16 weeks of HFDs, the researchers found that CD1dKO mice were protected against obesity and NASH progression, and CD8^+^ T-cell-depleted mice were also protected from NASH while continuing to result in weight gain. In addition, increased CD8^+^ T-cells were also observed in human liver sections from patients with NASH. Therefore, these data suggest that under HFD feeding stress, NKT cells and CD8^+^ T-cells synergistically contribute to the inflammation and fibrosis related to NASH progression (28). To further explore the underlying mechanisms for the development of NASH and HCC, a recent study used a rodent mole of long-term choline-deficient HFD to recapitulate NASH and NASH-induced HCC in humans. The analysis revealed that NKT cells primarily lead to steatosis through secreted LIGHT (97). Interestingly, unlike molecular mechanism determining NASH development, the underlying mechanisms for the HCC development may involve hepatocellular LTβR and canonical NF-κB signaling, which facilitates the transition from NASH to HCC (97). Overall, several hepatocellular signaling pathways including lymphocytes, LTβR, and NF-κB facilitate HFD-driven liver cancer.

### Breast cancer

Human breast adipose tissue, a heterogeneous cell population, is composed of multipotent MSCs, mature white adipocytes, committed progenitor cells, immune cells, endothelial cells, and fibroblasts. Under the effects of external stimulation, these MSCs can differentiate into various cell types, such as adipocytes, fibroblasts, and myofibroblasts (98). It has been recognized that obesity can promote myofibroblast differentiation within mammary adipose tissue, contributing to the microenvironment fibrotic remodeling and breast cancer progression (99). Upon further investigations, the researchers found that myofibroblast differentiation was increased by HFDs via downregulating microRNA (miR)-140 expression in mammary adipose tissue through a TGF-β1/SMAD3/miR-130 negative-feedback loop. In particular, HFD-increased TGF-β1 signaling leads to activation of SMAD3, which binds to and suppresses miR-140. This blocks miR-140 from targeting SMAD3 for degradation, causing amplified TGF-β1/SMAD3 signaling and the downregulation of miR-140, thus promoting myofibroblast differentiation (100). *In vivo* and *in vitro* studies have shown that myofibroblasts and its induced fibrotic microenvironment are related to increased initiation and growth of breast cancer (99, 100). Overall, HFDs may increase the initiation and growth of breast cancer through miR-140 downregulation-dependent myofibroblast differentiation in human breast adipose tissue.

### Gastric cancer

Epidemiological evidence has demonstrated that dietary fat is a critical risk factor for gastric cancer, which is a malignant tumor that originated from gastric mucosal epithelial cells (101–103). However, little is known about how dietary fat influences the function of gastric mucosal cells and contributes to precancerous lesions and hence gastric cancer. One recent study showed that in the precancerous gastric mucosa, fat deposition was obtained in the gastric pits in mice exposed to a HFD (60% fat calories) at 12 weeks. Ectopic fat accumulation then led to the disruption of organelle homeostasis in the gastric mucosa, as evidenced by remarkable increases in the expression of LAMP2A (the lysosomal marker), COX IV (the mitochondrial marker), Calnexin (the ER marker), and GM130 (the Golgi marker) in the gastric mucosa at 20 weeks (104). Therefore, HFD feeding first leads to excess lipid incorporation in the stomach, and then causes the disruption of organelle homeostasis in the precancerous gastric mucosa. In addition, HFD promoted stem-cell divisions and the development of gastric malignancies in the gastric mucosa, accompanied by activation of β-catenin pathway. However, β-catenin activation and stem cell marker-positive cells were not observed in mice with no leptin signaling. Meanwhile, the PI3K pathway, the downstream of leptin receptor, was also decreased (104). These data suggest that leptin signaling regulates β-catenin signaling. Taken together, HFD-driven lipid accumulation and organelle biosynthesis dysregulation confer cancer stem cell-like properties to the gastric mucosa via leptin, PI3K, and β-catenin signaling pathways (104).

### Intestinal cancer

Existing evidence has linked a HFD to intestinal cancer (2, 105–107). For instance, a recently published article showed that a long-term HFD (60% fat diet, 9–14 months) contributes to the early intestinal tumorigenesis (108). Similar findings were obtained in another study, showing that HFDs could promote intestinal tumor progression in genetically susceptible *K-ras*^*G*12*Dint*^ mice in the absence of obesity but based on marked alterations in gut bacterial communities (109). Exposure to HFDs led to many intestinal adaptations: (i) intestinal villi became shorter, while intestinal crypt depth was increased (108, 110); (ii) the numbers of intestinal stem cells (ISCs) increased while those of Paneth niche cells decreased; (iii) ISCs could expand in the absence of signals from Paneth cells; (iv) progenitors became more stem-cell like, acquiring the capacity to form intestinal organoids. These adaptations may partially contribute to the development of early intestinal tumorigenesis and subsequently cancer (108). There are two well-defined mechanisms for increased cancer risk induced by HFDs. One is via enhancing stemness and tumorigenicity of intestinal progenitors through activation of PPARδ/β-catenin signaling cascade (108). Consistently, other studies also show that HFDs and sustained PPARδ activation are associated with colorectal cancer initiation and progression (107, 111, 112). However, inhibition of PPARδ signaling may exert modest anti-cancer effects (113). The other is through inactivation of nuclear bile acid (BA) receptor farnesoid X receptor (FXR), which resulted in BA deregulation and promoted colon cell proliferation (110). These data indicate that inhibition of PPARδ and activation of FXR after exposure to HFDs may blunt tumor initiation and progression. Future investigations are needed to address how HFDs induce activation of PPARδ signaling and inhibition of FXR signaling.

## Potential treatment options

The demonstration of a link between HFDs and metabolic disorders and certain types of cancer has prompted research into strategies to reduce HFD-driven diseases and cancer. Undoubtedly, lifestyle changes, such as improving dietary regiments and enhancing physical activity, are the most successful interventions. In this regard, dietary interventions aimed at decreasing circulating FFAs and limiting inflammation originated from alterations in gut microbial communities are attracting increasing clinical interest. Although still in its infancy, promising findings have been reported by several studies. Circulating FFAs levels are strongly decreased without causing ectopic lipid accumulation in HFD-fed mice with dietary supplementation of Atglistatin, a selective inhibitor of adipose TG lipase, accompanied by decreased weight gain, insulin resistance, and liver diseases (114). Preclinical studies have also reported a metabolic benefit associated with the anti-inflammatory impacts of targeting PPARγ (115), ERS (116), IKKβ/NF-κB (117), and shifts in gut microbial communities (118). For instance, HFD-fed mice treated with Lactobacillus plantarum KY1032 and Lactobacillus curvatus HY7601 exhibited decreased body weight, fat mass, and levels of circulating leptin, insulin, total cholesterol and liver toxicity biomarkers, accompanied by alterations in gut bacterial composition and diversity (118). In addition, markers of inflammation and obesity are reduced with dietary supplementation with amino acid (especially leucine) and plant components (such as luteolin and flavonoids), alone or in combination, as were performed in HFD-fed mice (46, 119–123). Although there are additional undefined approaches, the above-mentioned findings give the field a critical boost and may be translated into human studies, thus improving interventional strategies to stem the negative effects of HFDs.

## Conclusions and perspectives for future studies

The literature reviewed here suggests that FFAs and LPS have been established as independent factors aggravating inflammation during HFD-induced chronic diseases and certain type of cancer. Targeting these factors may provide effective approaches for therapies against associated metabolic perturbations. Although great strides have been made in animal models to understand the signaling pathways implicated in HFDs-induced chronic diseases and certain type of cancer, more effects should be made to confirm whether these signaling pathways are physiologically valid in humans, and additional transcription factors and target genes need to be identified to reduce the overwhelming burden of chronic diseases and cancer.

## Author contributions

YD and LZ wrote the manuscript, KX designed the manuscirpt, CZ, BS, FL, and XK revised the manuscript. All authors read and approved the final version of the manuscript.

### Conflict of interest statement

The authors declare that the research was conducted in the absence of any commercial or financial relationships that could be construed as a potential conflict of interest.
